# Initiatives to detect and prevent death from perioperative deterioration

**DOI:** 10.1097/ACO.0000000000001312

**Published:** 2023-09-28

**Authors:** Linda M. Posthuma, Benedikt Preckel

**Affiliations:** a Department of Anesthesiology and Intensive Care Medicine, Amphia Hospital, Breda; b Department of Anesthesiology, Amsterdam University Medical Centre, location University of Amsterdam; c Amsterdam Public Health Research Institute, Quality of Care, Amsterdam University Medical Centre, Amsterdam, The Netherlands

**Keywords:** cognitive aids, failure-to-rescue, family participation, postoperative visit, remote monitoring

## Abstract

**Purpose of review:**

This study indicates that there are differences between hospitals in detection, as well as in adequate management of postsurgical complications, a phenomenon that is described as ‘failure-to-rescue’.

In this review, recent initiatives to reduce failure-to-rescue in the perioperative period are described.

**Recent findings:**

Use of cognitive aids, emergency manuals, family participation as well as remote monitoring systems are measures to reduce failure-to-rescue situations. Postoperative visit of an anaesthesiologist on the ward was not shown to improve outcome, but there is still room for improvement of postoperative care.

**Summary:**

Improving the complete emergency chain, including monitoring, recognition and response in the afferent limb, as well as diagnostic and treatment in the efferent limb, should lead to reduced failure-to-rescue situations in the perioperative period.

## INTRODUCTION

Worldwide, each year over 4 million people die within 30 days after surgery [1]. Although the occurrence of postoperative complications and critical events is quite similar among hospitals, large variations between hospital mortality rates are still observed. This indicates that there are differences between hospitals in detection, as well as in adequate management of postsurgical complications, a phenomenon that is described as ‘failure-to-rescue’ [2,3].

The postoperative complication rate is conservatively estimated to be 18% [3]. In older patients, as well as in patients undergoing more complex surgery or nonelective surgery, these rates are even higher [4]. The Vascular Events in Noncardiac Surgery Patients Cohort Evaluation [5] study group demonstrated in a prospective cohort of 21 842 patients undergoing in-hospital noncardiac surgery that myocardial injury occurred in 17.9% of patients, major bleeding in 14.2% of patients and sepsis in 4.1% of the patients. These were the most frequently occurring postoperative complications and were significantly associated with 30-day mortality [5]. With an expected rise in high-risk complex surgical patients and procedures in the coming years, it is imperative to reduce the incidence of preventable morbidity and mortality.

**FIGURE 1:**
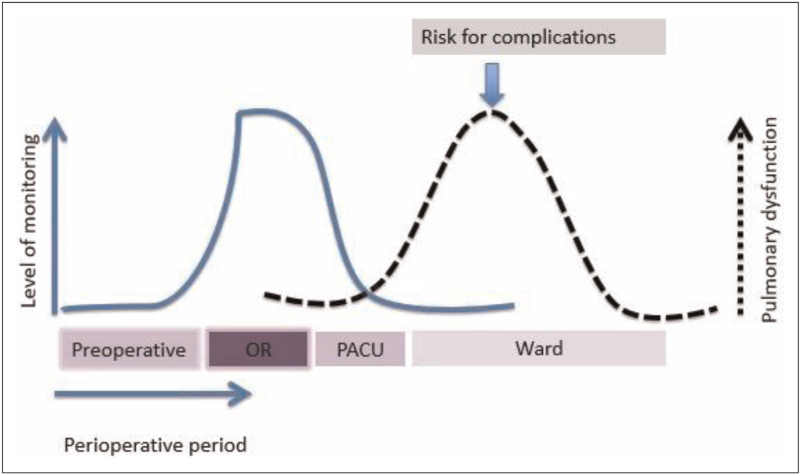
Development of complications and level of monitoring (previously published in [6]). While the level of monitoring is high during the time in the operating room (OR), it declines significantly during the stay of a patient on the post-anaesthesia care unit (PACU) and is low on the surgical ward, whereas possible complications have the highest incidence on the ward on days 2 and 3 after the operation.

Most postoperative complications occur in the first few days after surgery when the level of monitoring is significantly less than in the operation complex (Fig. 1) [6]. Initiatives to detect and prevent death from perioperative deterioration should therefore focus on this early postoperative period. In this review, recent initiatives to reduce failure-to-rescue in the perioperative period are described. 

**Box 1:**
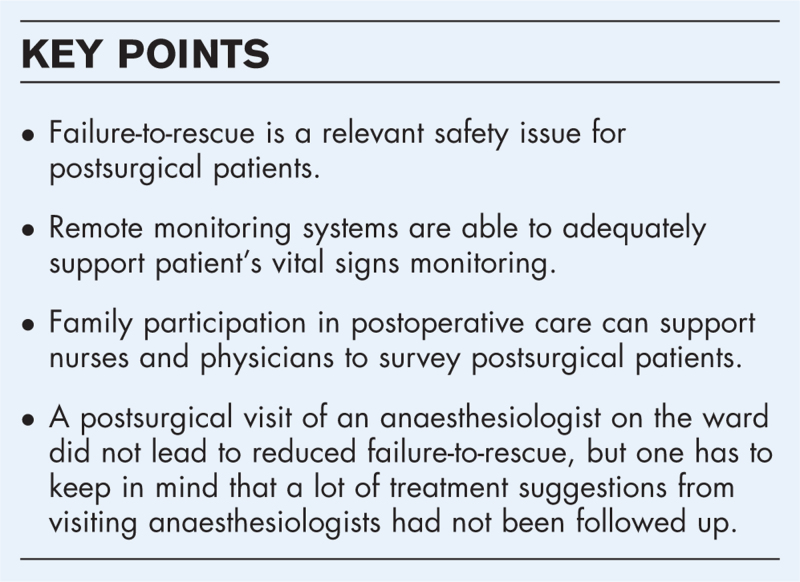
no caption available

## INITIATIVES TO DETECT AND PREVENT DEATH FROM PERIOPERATIVE DETERIORATION

### Complication prevention

The risk for perioperative complications is mainly associated with the patient's clinical conditions, while the surgical procedure itself only plays a minor role. Main surgical risk factors for development of postoperative complications are nonelective surgery, long duration of surgery, and the type of surgery. The role of anaesthesia is almost negligible, whereby – in general – there is no effect of type of intraoperative anaesthesia on the development of complications [4,7–9]. Because most postoperative complications are associated with preoperative patient morbidity, it is difficult to prevent the occurrence of complications during perioperative care in patients with significant comorbidity. However, some initiatives focus on perioperative risk reduction for occurrence of postoperative complications, and these will be described here first.

#### Cognitive aids

To prevent occurrence of complications or treat complications adequately, checklists and cognitive aids can help medical staff to remember all essential steps in an acute treatment plan or during a complex procedure. Currently, with an ever-increasing focus on patient safety and high quality of care [10], the use of checklists and cognitive aids, both inside and outside the operation theatre, are indispensable and gain increasing popularity [11–13].

Critical situations in the perioperative setting require rapid, effective and coordinated medical interventions. However, many events are rare and stressful, and thus, human errors easily occur, contributing eventually to failure-to-rescue [14]. Cognitive aids can be used individually to accomplish a complex multistep process, or they can be used in team settings to improve adherence to local protocols and guidelines in routine care or during emergency procedures [15–17]. Cognitive aids reduce the omission of critical steps, improve communication, teamwork and leadership, and contribute to a surgical safety culture [18^▪▪^]. Apart from their use in the operating theatre, cognitive aids are also introduced at the surgical ward [18^▪▪^].

#### Family participation

Another initiative that might prevent occurrence of postoperative complications is family participation. Basic and fundamental postoperative care is essential in preventing postoperative complications [19,20]. In paediatric surgery, it is known that the presence of relatives reduces stress and anxiety, resulting in a better medical environment with less postoperative pain and faster recovery of the paediatric patients [21,22]. Patient safety programmes support family involvement [23–25], and involving relatives in medical fields outside the perioperative setting resulted in beneficial effects, for example improved patient outcome and reduced risk of hospital readmission [26,27]. Initiatives to activate patient relatives in perioperative care are emerging, whereby family involvement programmes are formed and installed at the adult surgical ward [19,28,29]. In a recently published small study [30], high-risk abdominal surgery patients in the intervention group participated in a family involvement programme, and this group was compared with patients in the control arm that received standard perioperative care. The family involvement programme consisted of extra information, task-oriented training, participation in basic care, attendance of relatives during ward rounds, and rooming in. The study results showed that patients in the intervention group mobilized longer periods of the day, and showed more adherence to breathing exercises, oral hygiene and cognitive activities, in comparison to the control group that did not participate in a family involvement programme. Patients, families and healthcare providers valued the respective programme positively. This initial small study of family participation in the perioperative setting showed that a family involvement programme is feasible [30]. Further studying this concept, also incorporating measurements on nursing workload and cost-effectiveness, is warranted.

### Early detection of patient deterioration

It is customary to distinguish between *failure-to-detect*, which is the time elapsed since a complication became detectable and initiation of any respective treatment, and the *failure-to-treat*, which is the failure to adequately treat a respective complication. In current clinical practice, detection of complications still relies on the intermittent (every 6–12 h) assessment of vital signs and patient condition by nursing staff [31]. As a result of this low frequency of monitoring, deterioration of patients on the general ward may go unnoticed for prolonged periods of time [32]. In most cases, measurable changes in vital signs could have identified patients at risk already hours before [33^▪^]. One day prior to intensive care admission or the occurrence of a serious adverse event, the modified early warning score is elevated, and increasing further as time to the serious adverse event or intensive care admission decreases [34].

Apart from the intermittent nature of vital signs measurement, the nurse-to-patient ratio on the ward is substantially lower than in post anaesthesia care units [35] and compliance of ward staff to vital signs measurement is low, especially when vital signs need to be measured more frequently [36].

Another shortcoming of manual intermittent vital signs measurement by ward staff is the inaccuracy of respiratory rate measurement, one of the vital signs that is associated most with clinical deterioration [37]. This vital sign is often guessed or repeated from previous recordings [38,39].

The low nurse-to-patient ratio and intermittent, delayed or inaccurate vital sign measurement may result in failure-to-detect and thereby contribute to otherwise preventable cardiopulmonary arrest and admission to the intensive care unit [40].

#### Remote monitoring

A highly popular and interesting initiative on the surgical ward that can overcome the shortcomings of manual intermittent vital sign measurements are remote monitoring systems [41]. For remote monitoring, a sensor, often a patch, is attached to the patient. The sensor measures one or several vital sings frequently, and these measurements are sent to a central computer. A notification to the nurse's pager system is sent when the vital sings exceed a preset threshold for a preset duration of time.

Feasibility studies showed that these systems measure vital signs continuously and accurately, without restricting patient mobilization [42,43]. Continuous wireless monitoring systems improve patient monitoring on the ward and can contribute to an early recognition of patient deterioration [33^▪^]. Worldwide, there is an enormous and steadily increasing interest in remote monitoring systems [44], as these systems possibly improve perioperative care. Whether these systems actually result in improved patient outcome has not been tested in large randomized controlled trials [45,46]. Recent literature does show improvement in patient outcome in postoperative patients in small randomized control, before-after or retrospective studies [47–50].

#### Establishment of vital sign ranges

Next to adequate patient monitoring, registration and interpretation of vital signs is necessary to early recognize and treat patient deterioration. The ward staff determines whether the measured vital signs are out of normal limits. However, normal ranges of vital signs in postoperative patients are frequently not adequately established. Normal limits of vital sing values vary between different early warning scoring systems. For instance, the zero-weighted score of the ‘National Early Warning Score’ (NEWS) is 12–20 breaths per minute and the zero-weighted score of the ‘Modified Early Warning Score’ (MEWS) is 9–14 breaths per minute. These differences can contribute to afferent limb failure [51]. A first initiative to establish a normal range of respiratory rate in postoperative patients showed a range of 13–23 breaths per minute as normal in this patient population [52]. A multicentre study using remote monitoring data can help establish normal and realistic ranges for all vital signs in postoperative patients, thereby reducing failure-to-detect.

Apart from the establishment of normal vital sign ranges in postoperative patients, re-evaluation of early warnings scores is an important step towards adequate patient surveillance. The early warning scores MEWS and NEWS are used most often. How the cut-offs point of the MEWS were established is not described in literature and this score is validated mostly in medical patients without evaluation of the normal values. The NEWS is extensively validated and had greater ability to discriminate deteriorating patients. A few trials including surgical patients were used in the establishment of the score, but there was no specific focus on the postoperative period. The burden of vital signs measurements for nursing staff, miscalculations of EWS and eventually delayed rapid response team activation, so called afferent limb failure, are frequently discussed topics in the recent literature. Therefore, new early warning scores that automatically generate alarms, specifically for use of remote monitoring systems and established in surgical patients, are currently developed and highly warranted [53^▪▪^,^54^^▪▪^]. The first initial studies in this field show promising results, for example Van der Sam *et al.*[53^▪▪^] recently showed that the diagnostic value of an early warning score based on continuous monitoring could detect the same amount of deteriorations as the MEWS in abdominal surgery patients, whereby manual vital sign measurements were unnecessary.

#### Artificial intelligence and alarms systems

Automatically generated alarms by remote monitoring systems, without causing too many false alarms, can help ward staff in recognition of patient deterioration. Many wireless monitoring systems use standard alarm settings whereby a notification is sent to the ward staff in case a vital sign crosses a preset alarm limit for a certain amount of time. These preset alarm limits are often based on the MEWS or NEWS, with several limitations as described above. Also, factors that influence vital signs, like patient characteristics (personal factors) or physical activity (situational factors), are usually not taken into account, with the consequence that automatic alarms can produce numerous false alarms -- and consequently alarm fatigue -- of ward staff [55]. On the opposite, permitting only a small amount of alarms may result in decreased sensitivity of patient deterioration detection. Both situations can result in failure to rescue even though continuous monitoring is applied [56]. Alternative alarm strategies incorporating situational and personal factors improve alarm precision and reduce the amount of false alarms [57^▪^]. Further improvement off alarm strategies to reduce total alarm rates as well as false alarm rates, and improving alarms for early detection of adverse events is an essential step in future optimization of remote monitoring systems [54^▪▪^]. Smart alarms might be created by the use of big data, machine learning, advanced databases, imputation techniques for missing data and integrated approaches of technology in the workflow of ward staff (nurses and doctors) [58^▪^,^59^,^60^].

However, worth mentioning, apart from the use of all these advanced technology approaches, false alarms will always continue to exist and a culture change for handling these newly alarm strategies on the ward is unavoidable [61].

#### Cardiac biomarker monitoring

A different way of patient monitoring for detection of patient deterioration is cardiac biomarker monitoring. In some patients, postoperative myocardial infarction goes unnoticed because of absent clinical features. The European Society of Cardiology [62] recommends cardiac biomarker testing in patients undergoing intermediate-risk and high-risk surgery, although this still is not common clinical practice.

The incidence of postoperative cardiac complications varies between 0.4% [8] for myocardial infarction and 18% [63,64] in case of myocardial injury, with mortality rates of 15% or higher [8]. Elevated cardiac biomarkers in the postoperative setting are associated with increased mortality, also when other ischemic features are absent [5]. Because of a lack in postoperative cardiac biomarker surveillance in noncardiac surgical procedures, perioperative myocardial infarction is often underestimated. Postoperative biomarker testing and anaesthesiology visits result in early detection of myocardial injury [65^▪^]. Postoperative cardiac evaluation [66] and anticoagulation therapy [67] are associated with lower mortality rates. Recently, a multicentre study in The Netherlands was started, investigating perioperative biomarker response in surgical patients. In this trial, incorporating 5000 high-risk surgical patients that undergo cardiac, colorectal, vascular and lung surgery, the levels of biomarkers PCT, CRPhs, IL-6, GDF-15, sFLT, NT-proBNP, cTNThs, CysC and NGAL are studied in an attempt to construct a perioperative prediction model for postoperative complications [68]. Perioperative biomarker testing is a recommendable initiative to reduce failure-to-detect as well as failure-to-rescue, as it may provide an objective and reliable risk stratification tool to guide treatment decisions.

### Adequate treatment of complications

#### Rapid response systems and teams

**FIGURE 2:**
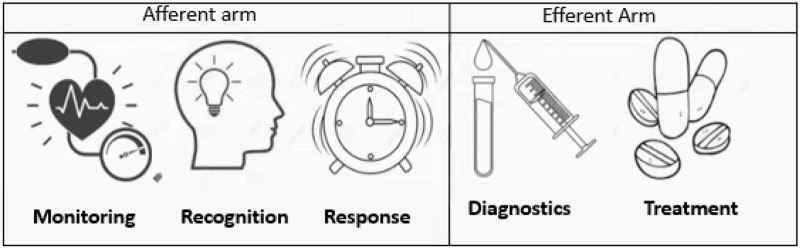
The afferent and efferent arm of a Rapid Response System. The afferent part of this emergency chain contains aspects for monitoring of vital signs and patient's wellbeing, recognition of deviations and adequate response to the deviation. The efferent part consists of indicated diagnostic procedures as well as adequate treatment (medication, interventional of surgical procedures and so on).

*Rapid Response Systems* (RRS) are commonplace in most hospitals and provide a safety net to allow for adequately handling deteriorating patients experiencing postoperative complications [69]. These systems include an afferent arm and an efferent arm [69], with the afferent arm focusing on the recognition of patient deterioration. The efferent arm reflects the establishment of an adequate diagnostic and treatment plan (Fig. 2). Both arms contain a complex series of elements. To adequately detect and treat postoperative complications to finally improve patient outcome, all elements of the RRS must function properly. The afferent arm can be improved by remote and cardiac biomarker monitoring, as described above.

The efferent arm frequently includes a rapid response team that evaluates a deteriorating ward patient quickly, to establish an efficient diagnostic and treatment plan. The effect of rapid response teams on patient outcome are less intensively studied than in the afferent arm [70,71]. Defining clinical outcome parameters is a challenge, as for example ICU admission can reduce failure-to-rescue, but can also be considered as an undesired event. Also, new ‘do-not-resuscitate’ orders are often discussed in respective patients. Although re-studying the effect of established RRS is not possible dur to ethical reasons, new initiatives are deployed recently, and will be described in the next chapter.

#### Multidisciplinary management

In multidisciplinary management, most likely the surgeon is responsible for the postoperative treatment of patients, together with other medical specialists, for example an intensivist or anaesthesiologist, instead of consulting a medical specialist with a specific request. Traditional consultations are often performed with significant delay, and follow up of the treatment recommendations is even more postponed. The ‘routine postsurgical anaesthesia visit to improve patient outcome’ (TRACE) [72^▪^] trial was a stepped-wedge randomized controlled multicentre trial that investigated whether regular visits to surgical patients on the ward by an anaesthetist (in training) on postoperative day one and three was able to reduce postoperative complications and mortality. A total of 5190 patients were eligible for analyses, 2490 patients in the control group and 2700 patients in the intervention group. On postoperative day one, 437 (16%) patients received extra recommendations by the anaesthesiologist and 293 patients on day three, respectively, but only 67 and 69% of these recommendations were followed up by the surgical staff. There was no reduction in 30-day mortality rate after a postoperative anaesthesia visit. Also, there was no difference in the incidence of postoperative complications between study groups, except a higher renal failure prevalence in the control group that received no anaesthesia visits. However, some findings are noteworthy to mention that might explain these mostly negative study results. The sample size calculation of the TRACE trial was based on the best available literature, and a mortality rate of 2% was expected. However, in the TRACE study, a mortality rate of only 0.5% was observed. Given this low mortality, a sample size of more than 20 000 patients would have been required to show a possible positive effect of the intervention. Furthermore, the study showed that there was still room for improvement as could be seen by the significant number of recommendations made by the anaesthesiologist: a substantial part of these recommendations was not followed up by ward staff. For future research, it is recommended that the postoperative anaesthesia visit should be embedded into routine surgical rounds, facilitating adequate effectuation of anaesthesiologist's recommendations [73].

Another initiative is the use of anaesthesiologist-intensivists for treatment of high-risk patients in the perioperative setting. Anaesthesiologist-intensivists are used to work with surgical patients and to work in collaborative multidisciplinary teams. They are trained to intervene on deteriorating patients, also outside the ICU. Until now, these interventions are mostly reactive. Intensivists become more proactive by being involved in high-risk patients in consult services. Anaesthesiologist-intensivists can provide clinical guidance, education, leadership and added value towards improving postoperative patient outcomes [74,75].

## CONCLUSION

With an expected rise in high-risk surgical patients and complex procedures in the coming years, it is imperative to reduce the incidence of preventable morbidity and mortality. In this review, initiatives to improve perioperative care by reducing development of complications or improving early and adequate detection and treatment of postoperative complications were described. These new initiatives bring interesting and promising results for future perioperative care.

## Acknowledgements

*None.*

### Financial support and sponsorships

*Support from institutional resources only, no external sponsorship received.*

### Conflicts of interest

*L.M. Posthuma: none.*

*B. Preckel: previously member of the Advisory Board of Sensium Healthcare, United Kingdom.*

## REFERENCES AND RECOMMENDED READING

*Papers of particular interest, published within the annual period of review, have been highlighted as:*

▪ of special interest
▪▪ of outstanding interest
